# Supplementary figures and images for: Serum lipopolysaccharide neutralizing capacity in ischemic stroke
Source: PLoS One. 2020 Feb 21;15(2):e0228806. doi: 10.1371/journal.pone.0228806 (PMC7034831; doi:10.1371/journal.pone.0228806)

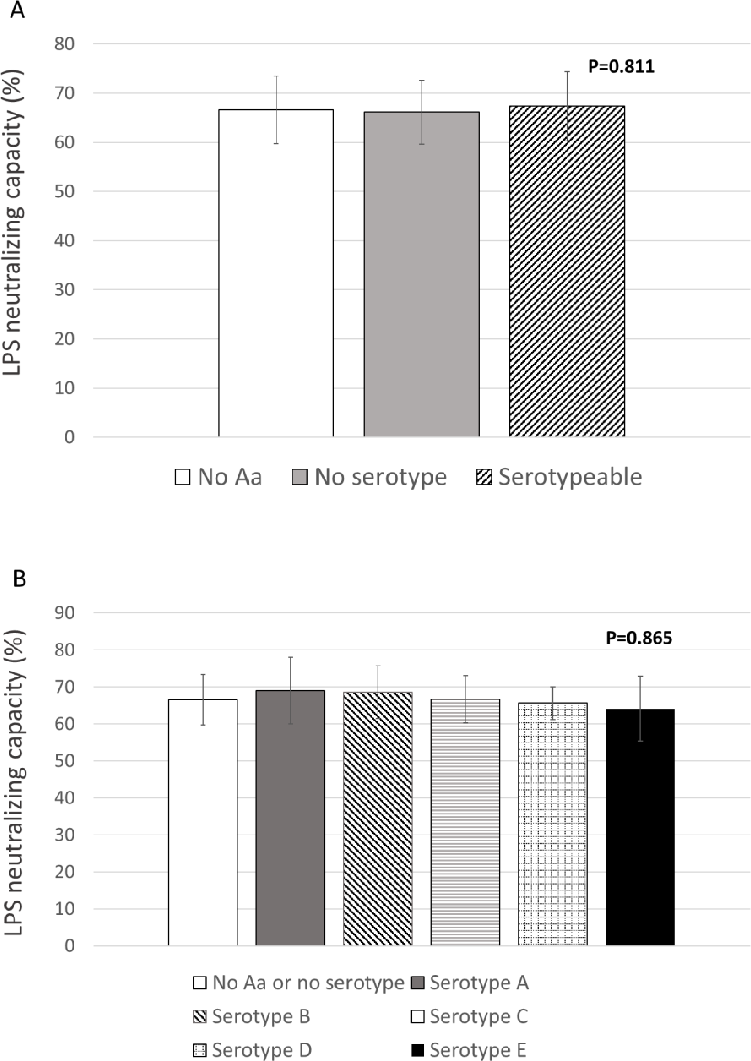

Supplement: S1 Fig — LPS-neutralizing capacity (LPS-NC) and A. actinomycetemcomitans determinations were performed among 198 subjects. The serotype (A to E) was determined by using qPCR on saliva samples. A) LPS-NC in subjects without the bacterium in saliva, with non-serotypeable bacterium strain, and with a serotypeable strain. B) LPS-NC according to different A. actinomycetemcomitans serotypes. Mean values are shown and the error bars present the standard deviation. P-value is calculated by using the ANOVA-test. (TIF) [file pone.0228806.s001.tif]
