# Supplementary material for: Serum lipopolysaccharide neutralizing capacity in ischemic stroke
Source: PLoS One. 2020 Feb 21;15(2):e0228806. doi: 10.1371/journal.pone.0228806 (PMC7034831; doi:10.1371/journal.pone.0228806)
Supplement: S1 Table — (DOCX) [file pone.0228806.s002.docx]

**S1 Table.**

|  | **Pearson correlation coefficient, p-value** | |
| --- | --- | --- |
| **Parameter of *P. gingivalis*** | **LPS (EU)** | **LPS-NC (%)** |
| Saliva concentration (genomes/ml)^1^ | -0.011, 0.880 | 0.004, 0.952 |
| Saliva IgA (Elisa units)^2^ | 0.072, 0.314 | -0.054, 0.457 |
| Saliva IgG (Elisa units)^2^ | 0.053, 0.461 | -0.019, 0.790 |
| Serum IgA (Elisa units)^2^ | 0.025, 0.723 | -0.025, 0.726 |
| Serum IgG (Elisa units)^2^ | -0.085, 0.236 | 0.115, 0.106 |

^1^ Analyzed by using qPCR; ^2^ analyzed by using multiserotype-ELISA, where the antigen is composed of three strains of killed *P. gingivalis* bacteria.
